# Technology Acceptance and Authenticity in Interactive Simulation: Experimental Study

**DOI:** 10.2196/40040

**Published:** 2023-02-15

**Authors:** Dahlia Musa, Laura Gonzalez, Heidi Penney, Salam Daher

**Affiliations:** 1 Department of Informatics Ying Wu College of Computing New Jersey Institute of Technology Newark, NJ United States; 2 Sentinel U Ellijay, GA United States; 3 College of Nursing University of Central Florida Orlando, FL United States

**Keywords:** health care simulation, interactivity, remote learning, video, technology acceptance, authenticity, nursing education, active learning, passive learning

## Abstract

**Background:**

Remote and virtual simulations have gained prevalence during the COVID-19 pandemic as institutions maintain social distancing measures. Because of the challenges of cost, flexibility, and feasibility in traditional mannequin simulation, many health care educators have used videos as a remote simulation modality; however, videos provide minimal interactivity.

**Objective:**

In this study, we aimed to evaluate the role of interactivity in students’ simulation experiences. We analyzed students’ perceptions of technology acceptance and authenticity in interactive and noninteractive simulations.

**Methods:**

Undergraduate nursing students participated in interactive and noninteractive simulations. The interactive simulation was conducted using interactive video simulation software that we developed, and the noninteractive simulation consisted of passively playing a video of the simulation. After each simulation, the students completed a 10-item technology acceptance questionnaire and 6-item authenticity questionnaire. The data were analyzed using the Wilcoxon signed-rank test. In addition, we performed an exploratory analysis to compare technology acceptance and authenticity in interactive local and remote simulations using the Mann-Whitney *U* test.

**Results:**

Data from 29 students were included in this study. Statistically significant differences were found between interactive and noninteractive simulations for overall technology acceptance (*P*<.001) and authenticity (*P*<.001). Analysis of the individual questionnaire items showed statistical significance for 3 out of the 10 technology acceptance items (*P*=.002, *P*=.002, and *P*=.004) and 5 out of the 6 authenticity items (*P*<.001, *P*<.001, *P*=.001, *P*=.003, and *P*=.005). The interactive simulation scored higher than the noninteractive simulation in all the statistically significant comparisons. Our exploratory analysis revealed that local simulation may promote greater perceptions of technology acceptance (*P*=.007) and authenticity (*P*=.027) than remote simulation.

**Conclusions:**

Students’ perceptions of technology acceptance and authenticity were greater in interactive simulation than in noninteractive simulation. These results support the importance of interactivity in students’ simulation experiences, especially in remote or virtual simulations in which students’ involvement may be less active.

## Introduction

### Background

The COVID-19 pandemic has prompted many health care providers to transition to remote or virtual simulations to comply with physical distancing guidelines. Many instructors opted to use commercial simulation software such as vSim for Nursing [1], Shadow Health [2], and Lippincott Clinical Experiences [3]. These software products have been valuable resources for health care instructors during the pandemic [4] and were appreciated by students [5]; however, flexibility is limited as these products are typically predeveloped and offer few options for customization. This creates challenges for instructors when the predeveloped scenarios do not meet the institution’s learning objectives. Some companies offer to modify their existing content or develop new scenarios according to requested specifications; however, these services often come at a high cost and are time-consuming. Many instructors who sought a more flexible and cost-effective modality used tele-simulation [6]. In tele-simulation, the instructor uses a videoconferencing platform to demonstrate a mannequin-based simulation to students remotely [7]. Tele-simulation has been shown to be beneficial for learning and well received by students [8], but the logistics of conducting a tele-simulation are difficult to orchestrate, especially during the pandemic [9]. Instructors often host the tele-simulation from a simulation facility and may need to assemble additional computer equipment to connect with students via the videoconferencing platform. As an alternative to tele-simulation, many instructors have found that simply recording their simulation videos was more feasible and cost-effective during the pandemic [10,11]. Similar to tele-simulation, simulation videos may require instructors to access simulation facilities; however, the recorded videos can be used to conduct numerous simulations without returning to the facility. A disadvantage of simulation videos is that interactivity is reduced compared with modalities such as tele-simulation. While watching videos, students’ engagement is passive, and they have minimal opportunity to collaborate or play an active role in the scenario.

### Objective

In response to the need for a virtual simulation technology that is flexible, cost-effective, and interactive, we developed a software that transforms multimedia content (eg, video, images, and text) into an interactive simulation that can be conducted remotely or locally. In a previous study, we found that our interactive video simulation (IVS) software promoted higher-order learning and authenticity to a greater extent than noninteractive simulation videos when used remotely over a videoconferencing application [12]. The IVS software can also be used in the classroom as a modality that reduces physical contact and engages students in an interactive and team-oriented experience. As a continuation of our prior work, this study investigated the role of interactivity in local simulations. We asked two research questions and two exploratory questions as follows:

1. Question 1: Is technology acceptance greater for interactive simulation than that for noninteractive simulation?
2. Question 2: Is interactive simulation perceived as more authentic than noninteractive simulation?
3. Exploratory question 3: Is technology acceptance of interactive simulation greater when the content is delivered remotely over internet than when it is delivered locally without internet?
4. Exploratory question 4: Is authenticity of interactive simulation greater when the content is delivered remotely over internet than when it is delivered locally without internet?

## Methods

We conducted an interactive video condition (INT) simulation and a video condition (VID) simulation to evaluate the role of interactivity in health care simulations. The INT simulation was conducted using a software that we developed. The methods used in this study are further discussed in this section.

### Development

#### IVS Software

We developed the IVS software in Unity 3D using the C# programming language [13]. The IVS software requires 2 monitors to be connected to the computer. One monitor displays a dashboard of buttons that are used by the facilitator to control the simulation content displayed on the second monitor. The dashboard is viewed only by the facilitator, and the second monitor displaying the content is viewed by the students. Each button on the dashboard corresponds to one piece of multimedia content, such as a video clip, image, or text. When a button is clicked on the dashboard, the corresponding content is displayed on the students’ monitor. The dashboard enables content to be displayed on the students’ monitor seamlessly and in any order. The multimedia content is imported into the software before the simulation. The software stores the content information and button data (eg, labels, colors, and order) in csv files. These files can be modified to assign content to buttons and to change the layout and design of the dashboard. During the simulation, the facilitator provides students with a Scenario, Background, Assessment, and Recommendation (SBAR) and asks them to describe the steps of the patient care. As the students describe their patient care, the facilitator displays the associated multimedia content on the students’ screen. For example, if students explain that they want to administer nitroglycerin medication, the facilitator will play the video clip of a nurse administering the medication. If students want to review the patient’s electrocardiogram, the facilitator will display an image of the electrocardiogram. When a button is clicked, the data are written to a log that the facilitator can later review to evaluate students’ performance.

The IVS software can be used to conduct a simulation locally or remotely. In a remote simulation, the facilitator connects with students via a videoconferencing application. The facilitator then uses the screen sharing feature to allow students to view the monitor displaying the simulation content, whereas the other monitor displaying the dashboard remains visible only to the facilitator. In this study, the simulation was controlled locally without the use of a videoconferencing application or the internet. In a previous study, we used the IVS software to conduct a remote simulation over Zoom [12,14]. We found that streaming videos over Zoom caused a reduction in the frame rate, and the videos lagged on the students’ screens. Many students reported that the lagging videos were distracting to their learning experiences [12]. In this study, the simulation was conducted locally without the internet to eliminate this factor, allowing us to focus exclusively on interactivity.

#### Educational Component

Simulation scenarios were developed to complement didactic or classroom content. The scenarios addressed stroke and chest pain management, which are challenging topics referred to as high risk and low volume in clinical practice. Simulation-based experiences are used to reinforce important concepts. In both interactive and noninteractive simulations, students were evaluated against Quality and Safety Education for Nurses competencies. The Quality and Safety Education for Nurses competencies include assessment, intervention medication, intervention communication, evaluation, and safety [15]. These competencies comprise the knowledge, skills, and attitudes that each prelicensure learner must develop to be competent. The scenarios incorporated the elements of these competencies. The interactive simulation enabled students to be more actively engaged in these competencies compared with the noninteractive simulation.

#### Scenarios

##### Overview

We used 2 scenarios from the nursing curriculum at the University of Central Florida (UCF), designed by nursing educators at UCF. The scenarios described a patient exhibiting stroke symptoms and a patient with chest pain. In these scenarios, the students were required to consider safety precautions for the patient, assess the patient’s condition, and administer medications according to the protocol.

##### Stroke Scenario

In the stroke scenario, a patient named Vera Real presented with a cerebral vascular accident or stroke. Students began their interventions by ensuring the safety of the patient, and then they conducted a thorough neurological assessment to identify a hypertensive crisis. The patient’s signs of a stroke should alert students to administer the appropriate prescribed medications according to physician orders and then report the patient’s status to the physician. Laboratory results, radiological scans, and physician orders were provided to guide the students’ patient care decisions.

##### Chest Pain Scenario

In the chest pain scenario, a patient named Anne Marie complained of chest pain and anxiety. This scenario encouraged students to think critically, as they must determine whether the chest pain is the result of anxiety or a serious cardiac event. At the start of their patient care, students ensured that the patient was safe, and then they administered oxygen and appropriate prescribed medications for cardiac irregularities and anxiety. Students should then provide a report to the physician. Laboratory results, electrocardiogram images, and physician orders were provided to students for review.

#### Simulation Content

The video content used in this study was recorded at the UCF College of Nursing simulation laboratory. The videos showed a nurse performing the scenarios with a mannequin patient. A total of 40 video clips were recorded, with 18 (45%) video clips for the stroke scenario and 22 (55%) video clips for the chest pain scenario. Each video clip showed the nurse performing 1 step in the scenario, such as washing hands, administering medication, or calling the provider. The videos were recorded as clips so that they could be used in the IVS software. We created exemplar videos by concatenating these video clips in the order of the correct sequence of steps. The exemplar video for the stroke scenario played for 15 minutes, 10 seconds, and the exemplar video for the chest pain scenario ran for 16 minutes, 8 seconds. All the videos were in the MP4 format and had a frame rate of 30 frames per second and resolution of 1920×1080. The INT and VID simulations included the same video content in the form of both unordered video clips and an exemplar video. In the INT simulation, the video clips were incorporated into the IVS software, and in the VID simulation, the video clips were used to guide the debriefing. The exemplar videos were used in both the INT and VID simulations. Therefore, the students were exposed to the same video content twice in each simulation. In addition to the video content, we captured images of the provider orders, laboratory results, and scans reviewed by the nurse in the videos. These images were provided for students to view via the IVS software in the INT simulation and were used during the debriefing in the VID simulation. The INT simulation also included text content to display the patient’s vital signs during the simulation.

### Recruitment

The participants of this study were 32 third-semester undergraduate nursing students at the UCF College of Nursing. Participants were recruited through a course required in the nursing curriculum. Student participation in the simulation scenarios was mandatory as part of the course, but completion of the surveys for the study was voluntary. The incomplete data of 9% (3/32) of participants were excluded, resulting in the inclusion of data from 91% (29/32) of participants. Of the 29 participants, 24 (83%) participants were identified as female and 5 (17%) as male. Racially and ethnically, 38% (11/29) of participants were identified as Hispanic, 34% (10/29) as White, 24% (7/29) as Asian, and 3% (1/29) as West Indian. All (100%) the participants reported previous experience with simulation: 24 (83%) participants had experience with mannequins, and 26 (90%) participants had experience with virtual simulation.

### Procedure

#### Overview

The study procedure was approved by the Institutional Review Board before the study was conducted. The design of this study was within-participants. The INT and VID simulations were conducted locally on the UCF campus. Students participated in the INT and VID simulations, and each simulation included either the chest pain or the stroke scenario. Students who viewed the chest pain scenario in the INT simulation viewed the stroke scenario in the VID simulation, whereas those who viewed the stroke scenario in the INT simulation viewed the chest pain scenario in the VID simulation. Students were randomly allocated to eight 3-member teams and four 2-member teams for a total of 12 teams. Students remained in their teams for the duration of the simulations. The teams’ order of participation was counterbalanced to prevent order effects: 6 teams participated in the INT simulation first and 6 teams participated in the VID simulation first. Before engaging in the simulation, students were shown the SBAR for 3 minutes.

#### INT Simulation

##### Setup

**Figure 1:**
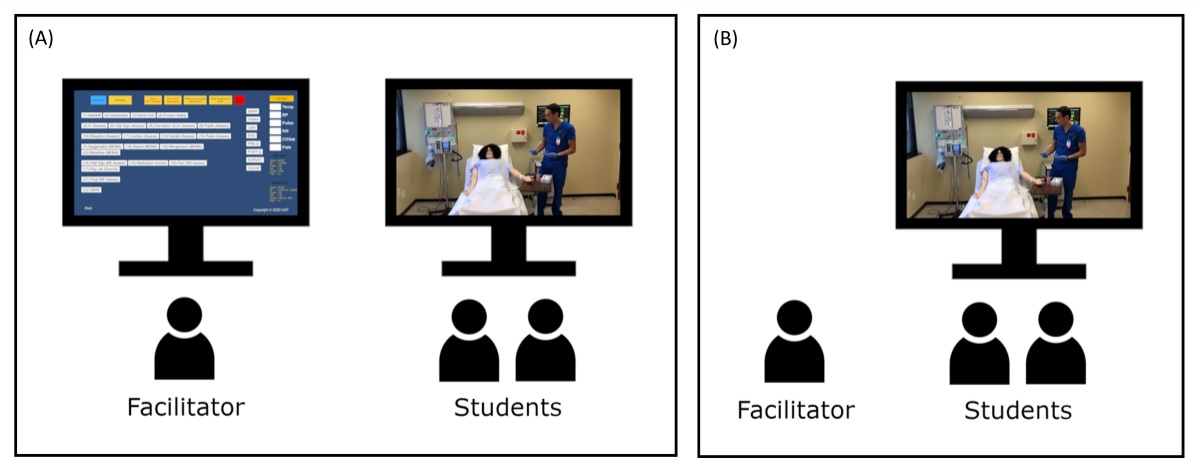
Setup for the (A) interactive video condition and (B) video condition simulations.

The INT simulation was conducted using the IVS software that we developed. The facilitator ran the software on a computer that was connected to 2 monitors. The dashboard was displayed on 1 monitor and remained visible only to the facilitator, whereas the students viewed the simulation content on another monitor. The INT simulation setup is shown in Figure 1A.

##### Procedure

**Figure 2:**
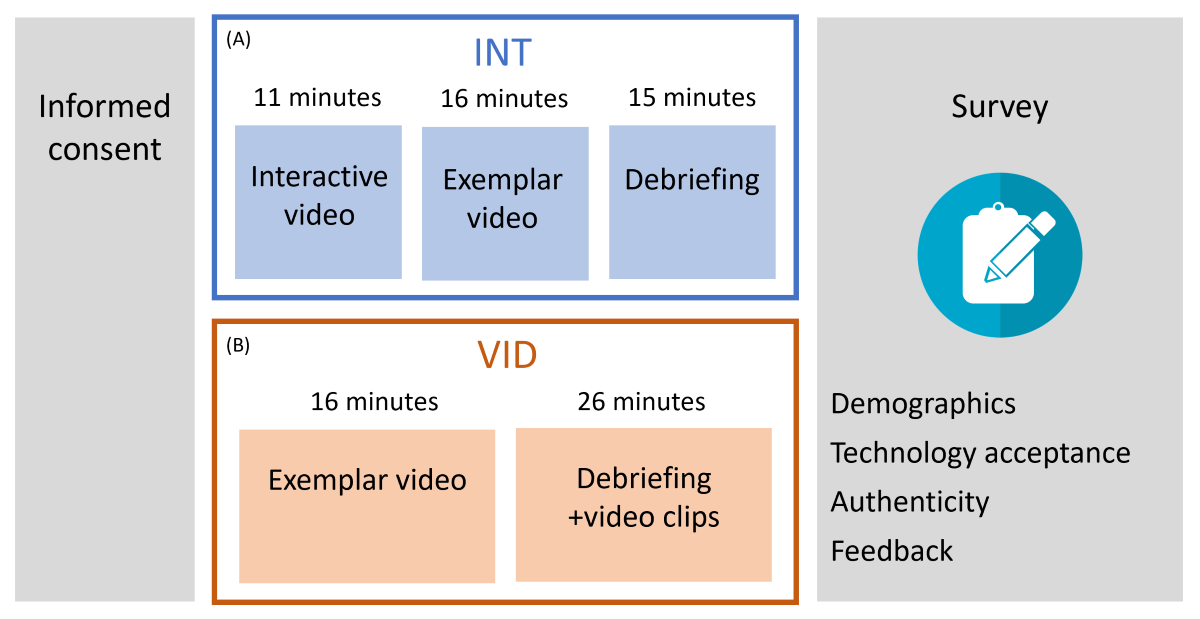
Procedures for the (A) interactive video condition (INT) and (B) video condition (VID) simulations. Informed consent was obtained at the start of each simulation, and students completed a survey at the end of each simulation.

The students participated in the interactive video via the IVS software for 11 minutes. During the interactive video, the facilitator asked the students to collaboratively describe the steps of their patient care. Students needed to unanimously agree on each step they would perform, and the facilitator then displayed the corresponding simulation content (ie, video clips, images, or vital signs) on the students’ monitor. If the students described a step not included in the simulation content, the facilitator acknowledged the students’ attempt and asked them to continue to the next step. Students could review the SBAR, provider orders, laboratory images, scans, or vital signs at any point during the simulation to inform their decisions. After completing the interactive video, the students watched the exemplar video for the scenario, which portrayed all the video clips in the correct sequence. The exemplar video was approximately 16 minutes long. The students were then debriefed by the facilitator for 15 minutes. In the debriefing, the facilitator discussed the students’ patient care decisions, recognized correct interventions, and clarified any areas of confusion or misunderstanding. After the debriefing, students were provided with a QR code to access a survey on their cell phones. The students completed the survey in 5 minutes. The procedure of the INT simulation is shown in Figure 2A.

#### Simulation

##### Setup

In the VID simulation, the students watched the exemplar video uninterrupted with no interactive components. Students viewed the correct sequence of steps that were performed by a nurse and did not provide their own input. The facilitator’s role in the VID simulation was to play the video for the students and conduct the debriefing. The setup of the VID simulation is shown in Figure 1B.

##### Procedure

Students watched the noninteractive exemplar video for approximately 16 minutes. After watching the video, the students were debriefed by the facilitator for 26 minutes. During the debriefing, the facilitator elaborated on the decisions made by the nurse in the exemplar video and responded to any of the students’ questions. Discussions during the debriefing were guided by video clips and images. After the debriefing, the students used a QR code to access a survey on their cellphones and completed the survey in 5 minutes. The procedure of the VID simulation is shown in Figure 2B.

### Measures

The measures evaluated in this study were technology acceptance and perceived authenticity of the simulations. Technology acceptance refers to the students’ willingness to use and adapt to a simulation technology, and authenticity refers to the extent to which a real-life encounter is accurately represented in a simulation. The survey used in this study included questionnaires derived from the Technology Acceptance Model (TAM) [16] and Virtual Patient Evaluation (VPE) [17] to measure technology acceptance and authenticity, respectively. The original TAM and VPE questionnaires are validated [16,17]; however, to make the questionnaires more suitable for this study, we modified or excluded some items. The TAM and VPE questionnaires were not validated after our modifications. The TAM questionnaire included 10 items scored on a Likert scale from *lower level* (1) to *higher level* (10). The TAM scores ranged from 10 to 100. The TAM questionnaire is presented in Textbox 1. The VPE questionnaire included 6 items scored on a Likert scale ranging from *strongly disagree* (1) to *strongly agree* (5). The VPE scores ranged from 6 to 30. Textbox 2 presents the VPE questionnaire. After the TAM and VPE questionnaires were completed, the survey included 3 open-response items to collect student feedback. The first item asked the students, “Which simulation technology did you prefer (video vs interactive video) and why?” The last 2 items asked the students, “Any comments about the simulation technology you just used?” and “Any other comments?” These data were used to quantify students’ preferences of the INT or VID simulations and to understand the factors that contributed to their preferences. All items of the survey were marked as required, except for the last 2 items, which permitted students to leave additional comments. The TAM questionnaire, VPE questionnaire, and student feedback questions were presented on different pages of the same survey. The survey was administered to participants via a QR code on Google Forms (Google LLC) [18]. The usability and technical functionality of the survey were tested before the study was conducted.

**Technology Acceptance Model (TAM) questionnaire that was included in a survey given to students after completing the interactive video condition and video condition simulations.**

**TAM1: learn**

- The use of the simulation software could help me to learn about nursing interventions more rapidly.

**TAM2: use**

- I think that I could easily learn how to use the simulation software.

**TAM3: time**

- The simulation software could help me get the most out of my time to learn about patients.

**TAM4: clarity**

- I believe that the learning carried out by the simulation software would be clear and easy to understand.

**TAM5: performance**

- The simulation software can improve my performance in patient care.

**TAM6: flexibility**

- I think that the simulation software is a flexible technology to interact with.

**TAM7: interesting**

- I find it interesting to use the simulation software for the learning about patients.

**TAM8: intention**

- I have the intention to use the simulation software when necessary to learn about patients.

**TAM9: clinical practice**

- The use of the simulation software may promote good clinical practice.

**TAM10: benefit**

- The use of the simulation software is beneficial for the care of my patients.

**Virtual Patient Evaluation (VPE) questionnaire that was included in a survey given to students after completing the interactive video condition and video condition simulations.**

**VPE1: decisions**

- While working on this case, I felt I had to make the same decisions a nurse would make in real life.

**VPE2: nursing care**

- While working on this case, I felt as if I were the nurse caring for this patient.

**VPE3: gathering info**

- While working on this case, I was actively engaged in gathering the information (eg, history questions, physical exams, lab tests) I needed to characterize the patient’s problem.

**VPE4: revising image**

- While working on this case, I was actively engaged in revising my initial image of the patient’s problem as new information became available.

**VPE5: summarizing problem**

- While working on this case, I was actively engaged in creating a short summary of the patient’s problem using medical terms.

**VPE6: nursing priorities**

- While working on this case, I was actively engaged in thinking about which findings supported or refuted my nursing priorities.

### Statistical Analysis

#### Overview

The participants’ scores for technology acceptance and authenticity were compared between the INT and VID simulations. Statistical analyses were performed using the Wilcoxon signed-rank test, which is a nonparametric test equivalent to the 2-tailed paired samples *t* test. We performed the statistical analysis of students’ total questionnaire scores to evaluate the overall perceptions of technology acceptance and authenticity. We also performed statistical tests on each questionnaire item to focus on the concept of each item separately. To prevent the occurrence of type I error in multiple comparisons, we applied the Bonferroni correction to adjust the error rate. An α value of .05 was assigned to the statistical tests. For the analysis of the TAM questionnaire results, the error rate was adjusted to .005 to account for 10 comparisons. To analyze the VPE questionnaire results, the error rate was adjusted to .008 to account for 6 comparisons. We also compared the participants’ technology acceptance and authenticity scores for the INT simulation from our previous study and this study. This analysis was performed using the Mann-Whitney *U* test, which is a nonparametric test equivalent to the 2-tailed independent samples *t* test. We used nonparametric tests because the data were not normally distributed; therefore, a parametric test is not recommended [19,20].

#### Data Exclusion

Missing and incomplete data from 3 participants were excluded. One participant did not submit the survey for either of the 2 simulations; 2 participants submitted the survey for only 1 of the 2 simulations. The Wilcoxon signed-rank test evaluates repeated measures; therefore, incomplete data could not be included.

### Ethics Approval

Ethics approval was granted by the UCF Institutional Review Board (ID: STUDY00002297). This study was approved with an exemption determination because it involved no or minimal risk to participants. Informed consent was obtained before students’ participation in the study. Students were informed that their deidentified survey data would be stored on a protected computer.

## Results

### Technology Acceptance

**Figure 3:**
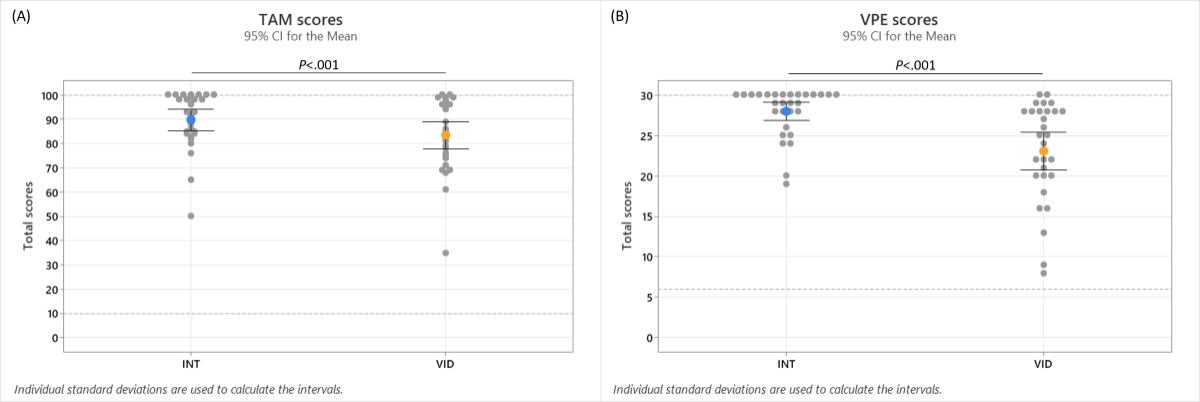
Students’ (A) Technology Acceptance Model (TAM) and (B) Virtual Patient Evaluation (VPE) scores for the interactive video condition (INT) and video condition (VID) simulations. The statistical data are shown in Table 1.

The students’ TAM scores ranged from 50 to 100 for the INT simulation and from 35 to 100 for the VID simulation. The mean TAM scores were 89.72 (SD 11.76) for the INT simulation and 83.38 (SD 14.89) for the VID simulation. The results were statistically significant for TAM scores of the INT and VID simulations (*P*<.001). The results for the TAM scores are shown in Figure 3A and Table 1. Comparisons between students’ INT and VID scores of individual TAM questionnaire items revealed statistical significance for TAM1 (*P*=.002), TAM3 (*P*=.002), and TAM9 (*P*=.004); these items pertained to learning, time, and clinical practice, respectively. Students’ mean TAM scores were higher for the INT simulation than for the VID simulation for all statistically significant TAM questionnaire items. The results for individual items of the TAM questionnaire are shown in Table 2.

**Table 1:** Results of the Wilcoxon signed-rank test evaluating students’ Technology Acceptance Model (TAM) and Virtual Patient Evaluation (VPE) scores for the interactive video condition (INT) and video condition (VID) simulations^a^.

Measure			W		*P* value		Effect size		Mean (SD)
**TAM**			302.00		<.001^b^		0.86		
	INT							89.72 (11.76)	
	VID							83.38 (14.89)	
**VPE**			293.00		<.001^b^		0.95		
	INT							27.97 (3.04)	
	VID							23.07 (6.18)	

^a^These data are represented as a graph in Figure 3.

^b^Statistically significant *P* values as defined by *P*≤.05.

**Table 2:** Results of the Wilcoxon signed-rank test evaluating students’ Technology Acceptance Model (TAM) scores for the interactive video condition (INT) and video condition (VID) simulations.

Question		W	*P* value	Effect size	Mean (SD)
**TAM1: learn**		172.00	.002^a^	0.81	
	INT				9.07 (1.22)
	VID				8.17 (1.67)
**TAM2: use**		64.00	.052	0.64	
	INT				9.03 (1.15)
	VID				8.48 (1.64)
**TAM3: time**		142.00	.002^a^	0.86	
	INT				8.79 (1.42)
	VID				7.83 (1.95)
**TAM4: clarity**		91.00	.015	0.73	
	INT				8.97 (1.27)
	VID				8.28 (2.00)
**TAM5: performance**		57.00	.035	0.73	
	INT				9.10 (1.29)
	VID				8.52 (1.75)
**TAM6: flexibility**		100.50	.094	0.48	
	INT				8.66 (1.74)
	VID				8.07 (2.27)
**TAM7: interesting**		85.00	.149	0.42	
	INT				8.93 (1.53)
	VID				8.55 (1.82)
**TAM8: intention**		151.50	.021	0.60	
	INT				8.72 (1.75)
	VID				8.10 (2.16)
**TAM9: clinical practice**		109.00	.004^a^	0.82	
	INT				9.31 (0.97)
	VID				8.72 (1.51)
**TAM10: benefit**		88.50	.020	0.69	
	INT				9.14 (1.38)
	VID				8.66 (1.52)

^a^Statistically significant *P* values as defined by *P*≤.005.

### Authenticity

The students’ VPE scores ranged from 19 to 30 for the INT simulation and from 8 to 30 for the VID simulation. The mean VPE scores were 27.97 (SD 3.04) for the INT simulation and 23.07 (SD 6.18) for the VID simulation. The results were statistically significant for the VPE scores of the INT and VID simulations (*P*<.001). The results for the VPE scores are shown in Figure 3B and Table 1. Comparisons between students’ INT and VID scores for individual VPE questionnaire items revealed statistical significance for VPE1 (*P*=.001), VPE2 (*P*<.001), VPE3 (*P*<.001), VPE4 (*P*=.003), and VPE6 (*P*=.005); these items pertained to decision-making, nursing care, gathering information, revising the image of the patient’s problem, and defining nursing priorities, respectively. The students’ mean VPE scores were higher for the INT simulation than for the VID simulation for all statistically significant VPE questionnaire items. The results for individual items of the VPE questionnaire are shown in Table 3.

**Table 3:** Results of the Wilcoxon signed-rank test evaluating students’ Virtual Patient Evaluation (VPE) scores for the interactive video condition (INT) and video condition (VID) simulations.

Question			W		*P* value		Effect size		Mean (SD)
**VPE^a^ 1: decisions**			158.00		.001^b^		0.85		
	INT							4.69 (0.54)	
	VID							3.79 (1.15)	
**VPE2: nursing care**			190.00		<.001^b^		1.00		
	INT							4.69 (0.47)	
	VID							3.55 (1.18)	
**VPE3: gathering info**			148.00		<.001^b^		0.94		
	INT							4.66 (0.72)	
	VID							3.76 (1.22)	
**VPE4: revising image**			87.50		.003^b^		0.92		
	INT							4.66 (0.72)	
	VID							3.93 (1.10)	
**VPE5: summarizing problem**			80.50		.013		0.77		
	INT							4.45 (0.91)	
	VID							3.93 (1.19)	
**VPE6: nursing priorities**			74.50		.005^b^		0.91		
	INT							4.83 (0.47)	
	VID							4.10 (1.24)	

^a^VPE: Virtual Patient Evaluation.

^b^Statistically significant *P* values as defined by *P*≤.008.

### Student Feedback

Of the 29 students who participated in this study, 28 (97%) preferred INT simulation and 1 (3%) preferred VID simulation. The student who preferred the VID simulation did not specify a reason but mentioned that although they preferred the VID simulation, they felt that they learned more in the INT simulation. Some of the students’ comments were given in the Textbox 3.

Students’ feedback indicated that they preferred the INT simulation over the VID simulation, primarily for reasons pertaining to critical thinking, knowledge retention, engagement, and enjoyment.

**Students’ comments regarding the simulations.**

**Comments regarding interactive video simulation**

- “[I] really liked the interactive video, a lot more than any other kind of simulation. Made me think critically and got to ask plenty of questions with instructor.”
- “Interactive video allowed me to make mistakes and learn from them, which I feel helps to solidify the knowledge.”
- “It felt live, even though it was on video.”
- “I loved the ‘choose-your-own-adventure’ style.”
- “It helped me learn how to prioritize nursing care. It felt more involved.”
- “I really like it for learning.”

**Comments regarding noninteractive video simulation**

- “It felt counterintuitive to watch a scenario unfold without me having a say in what happens.”
- “I found myself losing concentration while watching the video. The interactive video kept me engaged.”

### Exploratory Results

**Figure 4:**
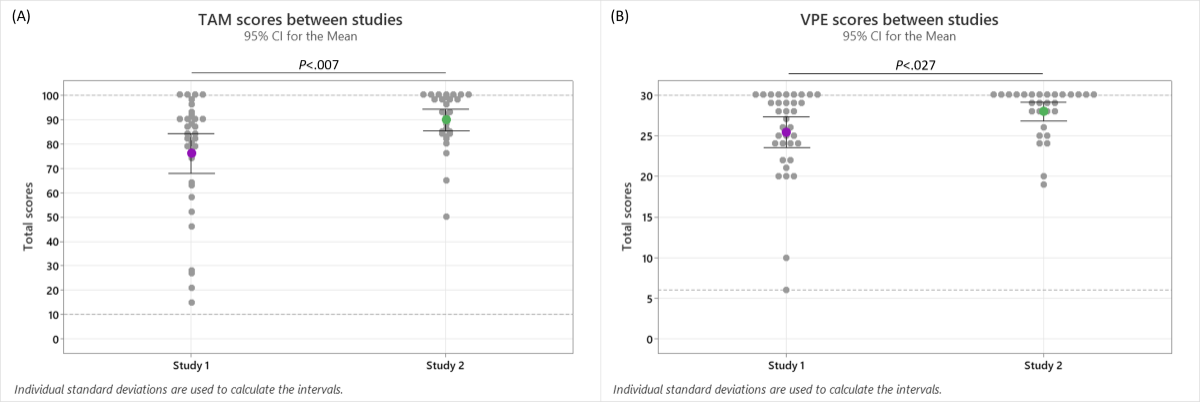
Students’ (A) Technology Acceptance Model (TAM) and (B) Virtual Patient Evaluation (VPE) scores between studies 1 and 2 for the interactive video condition simulation. The statistical data are shown in Table 4.

The exploratory analysis evaluated students’ TAM and VPE scores of the INT simulation from the first and second studies. Study 1 refers to our previous study [12] and study 2 refers to this paper. Students’ TAM scores ranged from 15 to 100 in study 1 and from 50 to 100 in study 2. The mean TAM scores were 76.06 (SD 23.60) for study 1 and 89.72 (SD 11.76) for study 2. The results were statistically significant for the TAM scores from studies 1 and 2 (*P*=.007). The TAM scores from the studies are shown in Figure 4A and Table 4. Students’ VPE scores ranged from 6 to 30 in study 1 and from 19 to 30 in study 2. The mean VPE scores were 25.43 (SD 5.51) for study 1 and 27.97 (SD 3.04) for study 2. The results were statistically significant for the VPE scores from studies 1 and 2 (*P*=.027). The results of the VPE scores from the studies are shown in Figure 4B and Table 4.

**Table 4:** Results of the Mann-Whitney *U* test evaluating students’ Technology Acceptance Model (TAM) and Virtual Patient Evaluation (VPE) scores between studies 1 and 2 for the interactive video condition simulation^a^.

Measure			W		*P* value		Effect size		Total participants, n		Mean (SD)
**TAM**			707.00		.007^b^		0.39				
	1							35		76.06 (23.60)	
	2							29		89.72 (11.76)	
**VPE**			667.50		.027^b^		0.32				
	1							35		25.43 (5.51)	
	2							29		27.97 (3.04)	

^a^These data are represented as a graph in Figure 4.

^b^Statistically significant *P* values as defined by *P*≤.05.

## Discussion

### Principal Results

Our results indicate that interactivity in health care simulation promotes students’ technology acceptance and perceived authenticity. Students also exhibited a strong preference for interactive simulation over noninteractive simulation.

#### Technology Acceptance

TAM predicts users’ acceptance of a technology by evaluating ease of use and perceived usefulness [21]. In the context of health care simulation, this implies that students are more likely to accept simulation technology if it is perceived to be uncomplicated and beneficial to their future learning. In this study, the students exhibited greater technology acceptance of interactive simulation than that of noninteractive simulation; that is, the interactive simulation technology was perceived by students to advance their learning (TAM1), be a valuable use of time (TAM3), and promote good clinical practice (TAM9). These results answer our first research question.

#### Authenticity

In the interactive simulation, the students were actively involved in the progression of the patient’s care and watched the case evolve based on their decisions. The interactive component of the simulation promoted a sense of agency in the scenario and reflected the role of a nurse more accurately. As a result, the students perceived the interactive simulation to be more authentic than the noninteractive simulation. The students felt responsible for the decision-making (VPE1) and care (VPE2) of the patient and were engaged in gathering information (VPE3), identifying the problem (VPE4), and determining priorities (VPE6). These results answer our second research question.

#### Student Feedback

Students largely preferred the interactive simulation over noninteractive simulation. Students reported that the interactive simulation increased their engagement, critical thinking, and knowledge acquisition and was overall more enjoyable. Interactivity was perceived to have broadly impacted many aspects of learning and was associated with positive outcomes.

### Exploratory Results

The exploratory analysis evaluated whether remote and local simulation modalities could impact students’ technology acceptance and perceptions of authenticity. The results indicate that local simulation may increase technology acceptance and authenticity compared with remote simulation. In our analysis, data for remote simulation were collected in our previous study, which was conducted over the internet via a videoconferencing application [12], and data for local simulation were collected in this paper. In both the studies, we measured technology acceptance and authenticity using TAM and VPE questionnaires. Our previous study was limited by poor internet connection, which caused the videos to lag over the videoconferencing application. The technology acceptance results in that study were not statistically significant and therefore not reported in our previous publication [12]. Students had mentioned that the lagging videos negatively impacted their simulation experiences [12], and we suspected that the poor internet connection contributed to the insignificant results. We decided to conduct this study to re-evaluate students’ perceptions of the simulations and eliminate any factors caused by poor internet connection. This allowed us to focus on the effects of interactivity on technology acceptance and authenticity more exclusively, without the results being obscured by uncontrolled variables. Our first study supported that interactive remote simulation promotes higher-order learning and increases authenticity compared with noninteractive remote simulation. This study demonstrated that interactive local simulation may further increase technology acceptance and authenticity compared with interactive remote simulation. While remote simulation has advantages, internet connection may introduce limitations that inhibit students’ experiences, in which case local simulation conducted without internet or with a more stable internet connection may be more advisable. These results answer our exploratory research questions.

These results are reported as exploratory and not definitive because there were minor discrepancies in the study procedures. In the first study, students in the INT simulation did not watch the exemplar video and students in the VID simulation did not view the video clips. Therefore, the students’ exposure to the multimedia content was unequal between the simulations. In this study, the students’ exposure to the content was equal in the INT and VID simulations. Our motivation for modifying the procedure was to improve the experimental design; however, this modification may have influenced the results of our analysis. A separate study focusing on evaluating remote and local simulations is required to provide definitive results. Nonetheless, this exploratory analysis provides further insight into remote and local simulation technologies.

### Limitations

Our study was limited by 2 factors. First, interactivity in multimedia education is formally defined as direct learner-computer interaction [22]; however, participants’ interaction with the IVS software in the INT simulation was indirect. In our study, students determined the system input (selection of the button representative of the patient care step) and were the recipients of the output (display of simulation content). Ultimately, it was the facilitator that directly interacted with the system by pressing buttons on the dashboard to prompt the display of content. The facilitator acted as a mediator between the students and the simulation system, resulting in indirect learner-computer interaction. However, despite the students’ indirect interaction, the INT simulation promoted a level of interactivity far greater than the VID simulation did. In the VID simulation, the students only passively watched the simulation video and provided no input. As a result, we believe that our comparison between interactive and noninteractive simulations remains valid. Direct learner-computer interaction in the INT simulation may have strengthened our results, but many of our comparisons between the INT and VID simulations remained statistically significant despite this limitation. We are currently developing the IVS software to permit direct learner-computer interaction, and we plan to conduct future studies to further investigate the role of interactivity in health care simulation. Second, our exploratory analysis compared the results for the INT simulation from this study and our first study published in [12], although the procedures of the studies were not the same. We modified the procedure of this study to equalize students’ exposure to the multimedia content between the INT and VID simulations because it was not equal in the first study. In the first study, the INT simulation did not include the exemplar video, whereas in this study, it did. Consequently, students had greater exposure to the content in this study than in the first study. Students’ higher TAM and VPE scores in this study may have been attributed not only to the local facilitation but also to the greater exposure to content. We included the analysis in this paper because it still has value, but we call it an “exploratory” analysis owing to this limitation. To confirm the validity of these results, we would need to conduct a future study in which the local and remote simulations incorporate the same procedure.

### Comparison With Prior Work

The role of interactivity in health care simulations has been addressed in previous studies. Medical education research often differentiates between passive learning and active learning. Passive learning implies a direct transfer of knowledge from the educator to the learner with minimal involvement from the learner, whereas active learning emphasizes engagement, observation, and reflection, and knowledge is constructed by the learner rather than transferred to them in active learning [23]. The advantages of active learning in students’ cognition have been supported by ample literature [24]. One meta-analysis of 225 studies found that active learning resulted in a 6% increase in students’ exam scores and failure rates were 55% higher in traditional lectures than in active learning classes [25]. Active learning has also been shown to promote long-term knowledge retention [26] and cultivate engagement [27]. After the onset of the COVID-19 pandemic, educators have used web-based infrastructure that can further facilitate active learning, promote knowledge acquisition, and improve learner satisfaction [28].

Despite the overwhelming endorsement of active learning, some educators are reluctant to implement these methods without more evidence-based research [29]. The results of active learning studies are often generalized without thorough evaluation of significant variables, such as the intensity of active learning, teacher and student characteristics, and outcome measures [30]. One study found that active participation did not improve students’ performance in simulation compared with passive observation and suggested that the debriefing structure may be the more influential factor [31]. Despite limited knowledge of the variables affecting active learning outcomes, the multiplicity of studies advocating active learning suggests that there must be some value in these methods. Active learning research is continuing to develop, and more critical analyses will enhance our understanding of active learning and its contribution to students’ experiences.

As we increased interactivity in our study, we observed a shift toward a nontraditional simulation structure. In traditional simulation, the debriefing is conducted after the simulation. Postsimulation debriefing involves providing minimal feedback during the simulation and discussing students’ performance after the simulation has been completed. In an alternative approach called Rapid Cycle Deliberate Practice (RCDP), the debriefing is a continuous process that occurs throughout the course of the simulation. The RCDP simulation is paused at various points to allow students to reflect on their decisions, discuss their subsequent tasks, and receive feedback from the facilitator. These reflective pauses are commonly referred to as microdebriefs. Previous research has demonstrated that microdebriefing reduces the cognitive load of the simulation by breaking it into segments that are more manageable for students to comprehend [32]. Learners have also reported that reflective pauses add greater value to their simulation experience than postsimulation debriefing [33]. In this study, the INT simulation incorporated a debriefing method resembling RCDP, whereas the VID simulation incorporated the traditional postsimulation debriefing. The use of segmented and itemized multimedia content in the INT simulation permitted the students to pause, reflect, and discuss at each step of the scenario. During these pauses, students collaborated among their groups to decide their next action, and the facilitator was present to guide their discussion. However, in a typical RCDP simulation, the facilitator immediately acknowledges students’ mistakes and allows them to rethink their actions. The INT simulation differed from the RCDP simulation in that the facilitator did not provide immediate corrections unless students described actions that were inappropriate for the scenario (eg, administering contraindicated medications). In these cases, the facilitator would address the mistake and let the students reconsider their decisions. However, if students missed or incorrectly ordered some steps, the facilitator proceeded with the simulation and discussed these mistakes after completion of the simulation. Productive failure pedagogy recognizes that there is value in allowing students to commit mistakes in simulations [34]. In this pedagogy, explicit instruction is avoided to allow students to execute their mistakes in a safe environment. Students’ mistakes are then discussed between the students and facilitators in a postsimulation debriefing. Productive failure has been shown to benefit students’ learning to a greater extent than explicit instruction [34]. The IVS software generates a simulation that combines the elements of both RCDP and productive failure. Reflective discussion is guided by the facilitator after each step of the scenario; however, students are not prevented from committing and learning from their mistakes.

### Conclusions

As the use of remote and virtual simulation technologies becomes more prevalent, the role of interactivity in students’ simulation experiences should be considered. This study demonstrated that interactivity in simulations may have advantages in terms of technology acceptance and authenticity. The interactive simulation in this study was met with greater technology acceptance and was perceived to be more authentic than the noninteractive simulation. Our exploratory analysis revealed that interactive simulation conducted locally without an internet connection may promote greater technology acceptance and perceptions of authenticity compared with remote delivery over an internet connection. Students also indicated a strong preference for interactive simulation over noninteractive simulation.

## Abbreviations

INT: interactive video condition
IVS: interactive video simulation
RCDP: Rapid Cycle Deliberate Practice
SBAR: Scenario, Background, Assessment, and Recommendation
TAM: Technology Acceptance Model
UCF: University of Central Florida
VID: video condition
VPE: Virtual Patient Evaluation

## Data Availability

The data are available upon request. Access to the data is currently restricted, as it may be used in future studies.
